# Macular changes after cataract surgery

**DOI:** 10.22336/rjo.2024.43

**Published:** 2024

**Authors:** Sorin Simion Macarie, Daniela Mariana Macarie

**Affiliations:** 1 “Iuliu Haţieganu” University of Medicine and Pharmacy, Cluj-Napoca, Romania; Cluj County Emergency Hospital, Cluj-Napoca, Romania,; 2 A.I.S.C.B.I. Cluj, Cluj-Napoca, Romania

**Keywords:** corneal melting, corneal anesthesia, neurotrophic keratitis, corneal foreign body, corneal perforation, BCVA = best corrected visual acuity, CME = cystoid macular edema, OCT = optical coherence tomography, SD-OCT = spectral domain optical coherence tomography

## Abstract

**Purpose:**

This article aims to highlight if the central macular retina suffers changes after cataract surgery, and to evaluate the eventually discovered changes regarding persistence.

**Material and method:**

This retrospective study, which lasted over three years (2021-2023), included patients who underwent cataract surgery performed by one surgeon. Spectral-domain optical coherence tomography (SD-OCT) imaging assessed macular changes and measured the central macular thickness.

**Results:**

A total of 240 eyes with diagnosed senile cataract were included in this study. The mean age was 66 years ± 4 years. Preoperative central foveal thickness was 210 ± 27.3 µm, the postoperative thickness on day 1 was 234.3 ± 40.2 µm, at 6 months 230.5 ± 35.2 µm, and the 1-year follow-up 229.2 ± 30.3 µm.

**Discussion:**

Macular changes after cataract surgery are easily confirmed by SD-OCT. The evaluation and monitoring of macular changes can be done by using central macular thickness assessment.

**Conclusions:**

The study provides data from a Romanian pool of patients. The values correlated well with those from similar studies of SD-OCT examinations, but differences were still observed, as there were different devices for performing SD-OCTs.

## Introduction

With aging, many patients experience one of the most frequent ophthalmic complaints, namely blurry vision. The main cause is cataract, a surgically treated pathology only [1]. Despite the technological advances that limit the number of complications, these still appear, with pseudophakic cystoid macular edema (CME) being the most frequent cause of unfavorable visual outcomes [2-4]. Optical coherence tomography (OCT) is the preferred method to study the retina and its changes with

spectral domain OCT being the most frequently employed. Literature is diverse regarding the results of macular changes after uncomplicated cataract surgery - some authors find an increase in retinal thickness [5,6], whereas others find it to be thinned [7].

Having such varied results, the present study aims to evaluate the impact of uncomplicated phacoemulsification surgery on macular thickness and bring its share of data to the existing pool.

## Materials and methods

This is a retrospective study, in which the data was collected for 3 years (2021-2023) in the Ophthalmology Clinic of Cluj-Napoca. Inclusion criteria were age 50 to 80 years, diagnosis of primitive cataract, presurgical retinal SD-OCT (Spectralis OCT, Heidelberg Engineering), and macula examination possible with normal aspect.

Exclusion criteria were any ocular comorbidity - trauma, ocular inflammations, previous retinal pathology of any type, intraoperative or early postoperative complications. Phacoemulsification was performed and a posterior chamber intraocular lens (IOL) was placed at the end of the surgery. Only one surgeon performed the surgery. When discharged, all patients were prescribed local steroid anti-inflammatory drugs for one month and follow-ups were scheduled on the 1^st^ day, at one month, six months, and one year. Spectral-domain optical coherence tomography (SD-OCT) imaging assessed macular changes, and the central macular thickness was measured and used as an indicator. Regarding statistical analysis, a paired t-test was used to compare eye measurements.

## Results

**Fig. 1:**
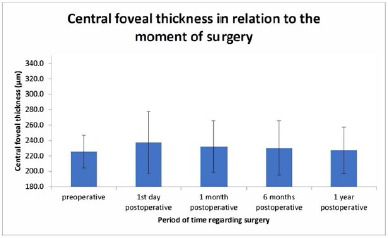
Central foveal thickness and moment of surgery

A total of 240 eyes with diagnosed senile cataract were included in this study. The mean age was 66 years ± 4 years. The preoperative central foveal thickness was 225.7 ± 21.3 µm. The postoperative thickness on day 1 was 237.3 ± 40.2 µm, at one month 232.3 ± 33.5 µm, at 6 months 230.5 ± 35.2 µm and at the 1-year follow-up 227.2 ± 30.3 µm, as observed in **Fig. 1** .

The difference between the preoperative and postoperative periods was statistically significant (p<0.05).

The mean preoperative best corrected visual acuity (BCVA) was 0.4 ± 0.1 using the Snellen chart. On the 1^st^ day after surgery it was 0.7 ± 0.2, at 6 months 0.8 ± 0.1, and after one year 0.9 ± 0.1. The difference between the preoperative and postoperative BCVA was statistically significant (p<0.05), between the 1^st^ postoperative day and at 1-year follow-up. All patients were assessed at the slit lamp using a +90D lens. On the 1^st^ day of follow-up, three patients had an elevated macula on clinical examination, and a macular thickness >273 µm on the SD-OCT examination, thus, they were additionally evaluated one week after surgery, when the macular thickness regressed.

## Discussion

There are several factors involved in the pathophysiology of the postoperative macular changes. Either due to free-radical release after surgical trauma, anterior segment ischemia, or light exposure of the retina in the postoperative period, Prostaglandin (Pg) production is linked to increased central macular thickness. This could justify prostaglandin production inhibitors [8-11]. Even though the incidence of macular edema is less than 7%, it is still one of the important risk factors for poor visual acuity results after cataract surgery [2,3,12].

In our study, the central macular thickness had increased during the follow-up period, with a maximum observed on the 1^st^ postoperative day. Afterward, a slow decline was observed. Studies by Nasreen et al. [13] and Kerner et al. [14] showed a similar trend regarding central macular thickness after uneventful phacoemulsification. Dad et al. study [2] showed an initial mild decrease on the first postoperative day. Similar results were obtained by Perente et al. [15]. It is thought that the light-scattering properties of the cataract play a role in the initial decrease in the optical quality of the OCT examination. In our study, vision was not impaired by the increase in retinal thickness and evolved as expected.

## Conclusions

Technological advances such as the OCT examination have made it possible to study patients in a non-invasive and cost-effective way to detect complications that if left untreated would significantly affect the patient’s quality of life. Limitations of this study included the retrospective design, which made it difficult to determine the causation between the studied processes. Further prospective studies are required to draw a pertinent conclusion.
